# The Hepatoprotective Effect of Leonurine Hydrochloride Against Alcoholic Liver Disease Based on Transcriptomic and Metabolomic Analysis

**DOI:** 10.3389/fnut.2022.904557

**Published:** 2022-07-07

**Authors:** Ke-Jia Wu, Pin-Pin Liu, Meng-Yuan Chen, Meng-Xin Zhou, Xin Liu, Qing Yang, Lin Xu, Zhiyong Gong

**Affiliations:** Key Laboratory for Deep Processing of Major Grain and Oil of Ministry of Education, Wuhan Polytechnic University, Wuhan, China

**Keywords:** leonurine hydrochloride, alcoholic liver disease, antioxidant, alkaloids, hepatoprotective

## Abstract

Excessive alcohol consumption can eventually progress to alcoholic liver disease (ALD). The underlying mechanism of ALD toxicity is primarily associated with oxidative damage. Many alkaloids have been reported to possess potential antioxidative efficacy, while the mechanism of their hepatoprotective activity against ALD is still not clear. In this study, eight alkaloids were selected from a monomer library of Traditional Chinese Medicine and evaluated for their antioxidant activity against ALD by the evaluation of Glutathione (GSH) and Malondialdehyde (MDA). The result suggested that Leonurine hydrochloride (LH) was a potent antioxidant that could reduce alcoholic liver damage. To further investigate the underlying mechanism of LH against ALD, the molecular pathway induced by LH was identified by RNA-seq analyses. Transcriptome data revealed the principal mechanism for the protective effect of LH against ALD might be attributed to the differentially expressed genes (DEGs) of PI3K-AKT, AMPK, and HIF-1 signaling pathways involved in the lipid metabolism. Given the hepatoprotective mechanism of LH is involved in lipid metabolism, the lipid metabolism induced by LH was further analyzed by UHPLC-MS/MS. Metabolome analysis indicated that LH significantly regulated glycerophospholipid metabolism including phosphatidylcholine, 1-acyl-sn-glycero-3-phosphocholine, phosphatidylethanolamine and 1-acyl-sn-glycero-3-phosphoethanolamine in the liver. Overall, this study revealed that the hepatoprotective mechanism of LH against alcoholic liver damage might be associated with the genes involved in glycerophospholipid metabolism.

## Introduction

Alcoholic liver disease (ALD) is caused by excessive drinking. In the early stages of the disease, triglycerides can accumulate in hepatocytes, increasing the risk of fatty liver. If alcohol consumption is continued, fatty liver can even progress to steatohepatitis or other chronic liver disease (1). A number of drugs have been reported as potential therapeutic agents for liver disease *in vitro* and *in vivo*, whereas their potential hepatotoxicity has hampered their application for ALD therapy (2). Disulfiram is an alcohol dehydrogenase (ADH) inhibitor, which is usually employed to treat alcoholism, while special care must be taken for its potential hepatotoxicity (3, 4). Anticraving drug (acamprosate) is a medication for alcoholism treatment and can prevent relapses of ethanol-dependent patients, whereas not suitable for all advanced ALD patients because of its hepatotoxicity (4). Thus, it is of great urgency to develop potential agents with low hepatotoxic activity against ALD.

The underlying mechanism of ALD toxicity is primarily associated with the changes in oxidation–reduction (redox) status caused by the oxidative metabolites of ethanol. Specifically, ethanol produces acetaldehyde and converts to acetate, while nicotinamide adenine dinucleotide (NAD) reduces to its reduced form (NADH) through the ADH pathway in liver cells. Meanwhile, excessive acetaldehyde and NADH produced by the oxidation of ethanol would result in stimulating lipogenesis and cause hypertoxicity (5–9). Therefore, restoring oxidative activity is a promising strategy for the treatment of ALD. Alkaloids exist in a variety of Traditional Chinese Medicine and display potential antioxidative efficacy with low hepatotoxic activity (10–12). Alkaloid tetrahydropalmatine was identified as a potent antioxidant by lipid peroxidation and hemolysis assays (13). Alkaloids—vindolicine III was isolated from Catharanthus roseus’s leaves and presented as a potential antioxidant against H_2_O_2_-induced oxidative damage in *cello* (14). However, the hepatoprotective activity of these reported alkaloids against ALD is not clear. In this study, eight alkaloids (purity >98.0%) were selected from the Library (Traditional Chinese Medicine Monomer Library, MedChemExpress, Shanghai, China) and evaluated for their antioxidant activity against ALD. We report thereon in this study the identification of leonurine hydrochloride (LH) as a potent antioxidant compound against alcoholic liver damage. To our knowledge, LH is firstly reported as a promising antioxidant compound against ALD *via* regulating the expression patterns of genes involved in the glycerophospholipid metabolism.

## Materials and Methods

### Reagents

Fetal bovine serum (FBS) was purchased from Gibco by Thermo Fisher Scientific Inc. (Shanghai, China). Penicillin– streptomycin solution was obtained from Genom (Hangzhou, China). Roswell Park Memorial Institute (RPMI) 1640 medium were purchased from Gibco by Thermo Fisher Scientific Inc. (Shanghai, China). DMSO was obtained from Sigma-Aldrich Inc. (Shanghai, China). The hepatocyte cells (LO2 cells) were obtained from Chinese Academy of Sciences (Cell Biology of Shanghai Institute, Shanghai, China).

### Cell Culture

The human hepatic cell line LO2 was derived from primary normal human hepatocytes and has been widely applied in human hepatocellular function studies, particularly those related to drug hepatotoxicity (15, 16). LO2 cell line was cultivated in 1640 medium with 1% penicillin (100 units/mL)/streptomycin (100 μg/mL) and 10% FBS at 37°C in a humidified atmosphere (95%) containing 5% CO_2_.

### 3-(4,5-Dimethylthiazol-2-yl)-2,5-Diphenyltetrazolium Bromide Assay

LO2 cells were seeded at 5,000 cells per well in a 96-well plate and incubated overnight at 37°C. The cells were treated with LH (0–500 μM) for 24 h (at least 6 replicates per group). Then 3-(4,5-dimethylthiazol-2-yl)-2,5-diphenyltetrazolium bromide (MTT) reagent was added to each well at a final concentration of 0.5 mg/mL for the additional 4 h. The medium was replaced with 100 μL DMSO. After shaking the plate for 10 min at room temperature in the dark, the viability of the cells was measured at 490 nm using a microplate reader.

### Glutathione Detection

To evaluate Glutathione (GSH) level in cell, GSH kit (Nanjing Jiancheng Bioengineering Institute, Nanjing, China) was utilized. The experiment was performed according to the manufacturer’s instructions. Briefly, 2.5 × 10^5^ cells were seeded in a 6-well plate, followed by culturing with ET (0.5%) for 24 h. After exposing the ethanol-treated LO2 cells to different alkaloids or LH at indicated concentrations for the additional 24 h, cells were harvested and washed twice with ice-cold PBS. Then the samples (at least three replicates per group) were processed according to the manufacturer’s instructions and immediately analyzed by Multi-Mode Microplate Readers. For preliminary screening, ethanol-treated LO2 cells were exposed to eight alkaloids (10 μM) for 24 h to study the antioxidative activity of different alkaloids against ALD. To study the dose-response experiment of LH on the alcoholic liver cell, ethanol-treated LO2 cells were exposed to LH (0–50 μM) for 24 h, followed by evaluating GSH level in cells.

### Malondialdehyde Detection

To evaluate Malondialdehyde (MDA) level in cell, MDA reagent kit (Nanjing Jiancheng Bioengineering Institute, Nanjing, China) was utilized. The experiment was performed according to the manufacturer’s instructions. Briefly, 1 × 10^6^ cells were seeded in a 6-well plate, followed by culturing with ET (0.5%) for 24 h. After exposing the ethanol-treated LO2 cells to different alkaloids or LH at indicated concentrations for the additional 24 h, the cells were harvested and washed twice with ice-cold PBS. Then, the samples (at least three replicates per group) were processed according to the manufacturer’s instructions and immediately analyzed by Multi-Mode Microplate Readers. For preliminary screening, ethanol-treated LO2 cells were exposed to eight alkaloids (10 μM) for 24 h to study the anoxidative activity of different alkaloids against ALD. To study the dose-response experiment of LH on the alcoholic liver cell, ethanol-treated LO2 cells were exposed to LH (0–50 μM) for 24 h, followed by evaluating MDA level in cells.

### RNA Extraction

A total of 1 × 10^6^ cells were seeded in a 6-well plate, followed by culturing with ET (0.5%) for 24 h. After exposing the ethanol-treated LO2 cells to LH (10 μM) for the additional 24 h, the cells were harvested and washed twice with ice-cold PBS. Then, total RNA was extracted from LO2 cells using Trizol reagent (Vazyme, Nanjing, China). After centrifuging at 12,000 × *g* for 20 min, the supernatant was collected and mixed with chloroform (200 μL). Afterward, the supernatant was kept for 20 min at room temperature and centrifuged at 12,000 × *g* for 20 min. The top water layer was transferred into a new tube and mixed with 0.5 ml isopropanol for 10 min at room temperature. After centrifuging at 12,000 × *g* for 10 min, RNA pellet was collected and resuspended in 1 ml of ethanol (75%), followed by an additional centrifugation at 8,000 × *g* for 5 min to obtain the final RNA sediment. RNA quality was determined by Nanodrop™ OneC spectrophotometer (Thermo Fisher Scientific Inc., Waltham, MA, United States). OD analysis revealed RNA yields of around 3.5–12.5 μg from 1 × 10^6^ cells.

### Quantitative Reverse Transcription PCR Assay

The gene expressions were monitored by RT-qPCR as described in our previous study (17). Specifically, RNA was converted to cDNA using PrimeScriptTMRT Master Mix (TakaRa, Japan). RT-qPCR was carried out using CFX96 Real-Time PCR Detection System (Bio-rad, Minneapolis, MN, United States). The thermal conditions were as follows: 95°C for 30 s, 35 cycles of 95°C for 5 s, 60°C for 30 s. The primers are presented in Supplementary Table 1. β-actin was used as an internal reference gene. The relative changes of mRNA expression were quantified by using the 2^–△^^△^ CT method (at least 3 replicates per group).

### RNA Library Preparation and Sequencing

A stranded RNA sequencing library was prepared according to manufacturer’s instructions by using KCTM Stranded mRNA Library Prep Kit (Catalog No. DR08402, Wuhan Seqhealth Co., Ltd., Wuhan, China) (18). Briefly, the mRNA was enriched by magnetic beads and broken into short fragments by fragment buffer. A random primer was used to synthesize a single strand of cDNA using the mRNA fragment as a template. After synthesizing and purifying double stranded cDNA, the terminus of the cDNA was repaired with base and added with sequencing adaptor. The fragment was caught by magnetic beads and amplified by T100 Thermal Cycler (BIO-RAD, United States). Qubit 3.0 with Qubit RNA Broad Range Assay kit (Life Technologies, Q10210) is used to quantify the cDNA library. Finally, the different cDNA library corresponding to 200–500 bps was sequenced on Illumina Novaseq 6000 sequencer (Illumina Inc., San Diego, CA, United States) with a pair-end 150 bp (PE150) model at Seqhealth Technology Co., LTD (Wuhan, China). The RNA-seq data were obtained based on two independent biological replicates. Each group includes two samples and all the samples were repeated for two times.

### RNA-Seq Analysis

Raw RNA-seq data were filtered by Trimmomatic (version 0.36). Statistical analyses were calculated by Student’s *t*-test to determine statistical significance between ET and ET + LH groups. Specifically, after discarding and trimming low-quality reads, clean data were assigned to the reference genome of Homo sapiens, based on the default settings of STRA software (version 2.5.3a). Reads mapped to the exon regions of each gene were counted by featureCounts (Subread-1.5.1; Bioconductor) and then RPKMs (Reads per Kilobase per Million Reads) were calculated. DEGs analysis were previously described (17). Briefly, DEGs were identified using the edge R package (version 3.12.1). The pathways with *p*-value < 0.05, *q*-value < 0.2 was considered significantly altered. Heat map was generated with an online website^1^ and Kyoto Encyclopedia of Genes and Genomes (KEGG) and Gene Ontology (GO) enrichment analysis were performed by KOBAS software (version: 2.1.1). Statistical analyses were calculated by Student’s *t*-test to determine statistical significance between ET and LH + ET groups.

### Liver Cells Metabolite Extraction

A total of 1 × 10^6^ cells were seeded in a 6-well plate, followed by culturing with ET (0.5%) for 24 h. After exposing the ethanol-treated LO2 cells to LH (10 μM) for the additional 24 h, the cells were harvested and washed twice with ice-cold PBS. Then, LO2 cells were extracted and mixed with methyl alcohol and methyl tert-butyl ether solution (1:2, V/V) for 60 s. The prepared cell solutions were dissolved with deionized water and kept for 1 h at room temperature. Finally, the solutions were centrifuged at 4°C for 20 min at 8,000 rpm.

### Ultrahigh Performance Liquid Chromatography-Tendem Mass Spectrometry (UHPLC-MS/MS) Analysis

The collected lipid metabolites of the cell samples (at least four replicates per group) were analyzed using QTRAP 6500 LC-MS/MS system (AB Sciex, United States) equipped with Acquity HSS T3 column (2.1 mm × 100 mm i.d., 1.8 μm, Waters, United States) as previously described (19). Specifically, the mobile phase solvent A: water/methanol/acetonitrile (1/1/1, v/v/v) containing 10 mM ammonium formate and 0.05% formic acid; solvent B: isopropanol. Flow speed: 0.4 mL/min. The column temperature was 45°C and the injection volume was 8 μL. The conditions used for the electrospray source were previously described (19). Specifically, the ion source temperature, 450°C; the ion spray voltage, 5,500 V in positive mode (5,500 V in negative model); the ion source gas I, 55 psi; the gas II, 60 psi; the curtain gas, 30 psi; the collision gas, medium; quantification of ion pairs corresponding to lipids by multiple reaction monitoring mode (MRM); detection widow, 60 s. According to our previous study, mass spectrometry information of the target lipid metabolites was prepared (19). Specifically, quality control (QC) sample was prepared by mixing equal amounts (50 μL) of each extracted sample. Moreover, the QC specimen was analyzed every four samples throughout the entire analysis procedure.

### Metabolic Pathway Analysis

Raw data were processed as previously described (19). Specifically, the quantification of target lipid metabolites was based on signal intensity relative to the corresponding internal standard by the multiple reaction MRM of triple quadrupole mass spectrometry. Peak alignment, retention time, and peak area extraction were performed using Analyst and SCIEX OS program. Principal component analysis (PCA), orthogonal partial least squares-discriminant analysis (OPLS.DA), and VIP analysis (VIP >1 and Fold Change Analysis <0.5 or Fold Change Analysis >1.5) were utilized to improve the analytical ability of the model and identify differences (20). Metaboanalyst 5.0^2^ was utilized to analyze the differential metabolites among the groups.

### Statistical Analysis

For statistical analysis, all data were analyzed with one-way analysis of variance (ANOVA) followed by the Dunnett’s method for multiple comparisons by using GraphPad Prism 5.0.

## Results

### Discovery of Leonurine Hydrochloride as an Antioxidant Against Alcoholic Liver Damage

**FIGURE 1:**
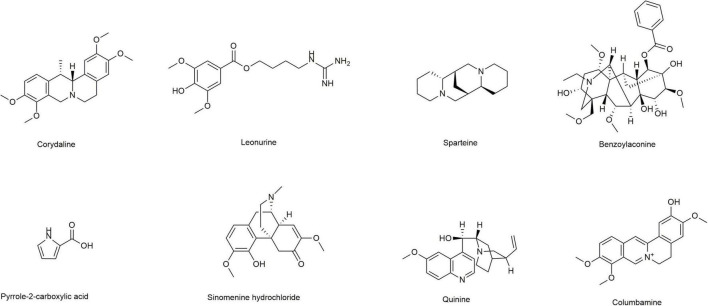
Chemical structures of different alkaloids.

**FIGURE 2:**
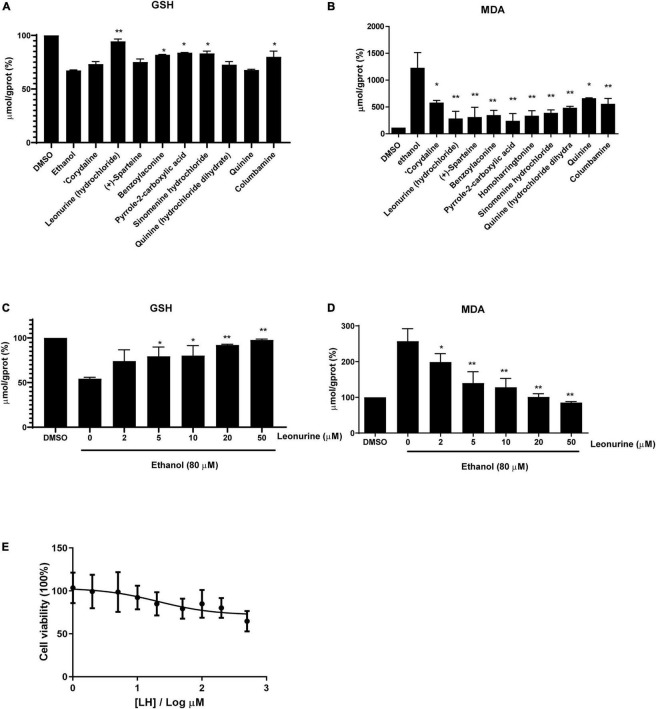
The antioxidative activity of different alkaloids (10 μM) against ethanol-treated LO2 cells. **(A)** The levels of Glutathione (GSH), **(B)** the levels of Malondialdehyde (MDA). **(C)** The levels of GSH in a dose-dependent manner (0–50 μM). **(D)** The levels of MDA in a dose-dependent manner (0–50 μM). The final result was normalized by protein concentration (mg prot/mL) and the unit of the final result was μmol/g prot. Values are expressed as mean ± SEM (*n* = 3). * and ** indicated a significant difference at *p* < 0.05 level, a highly significant difference at *p* < 0.01 level, respectively, between ET group vs. different alkaloid groups using the independent *t*-test. **(E)** Cell viability of LO2 cells after exposure to Leonurine hydrochloride (LH) (0–500 μM) for 24 h. Values are expressed as mean ± SEM (*n* = 6).

Induction of lipid peroxidation (MDA) and depletion of GSH have been implicated in the pathogenesis of ALD. In this study, we first selected eight alkaloids (Figure 1) from the Traditional Chinese Medicine Monomer Library (MCE, Shanghai, China) and evaluated their hepatoprotective activity (10 μM) against alcoholic liver damage by detecting GSH and MDA. From the preliminary screening results, LH, a natural alkaloid extracted from Herba leonuri, is suggested as a promising antioxidative damage agent (Figures 2A,B). The results showed that LH can restore the reduction of GSH and the induction of MDA caused by alcohol-induced liver oxidative damage. To further investigate the effects of LH on antioxidative activity and cytotoxicity, a dose-response experiment of LH on the alcoholic liver cell was performed. The results revealed that LH can induce the activation of GSH and suppress MDA releasing in a dose-dependent manner (Figures 2C,D). Meanwhile, the cell viability results revealed that LH displays less cytotoxicity (IC_50_ = 153.8 μM) on LO2 cells even at 500 μM (Figure 2E). It suggested that LH might suppress alcohol-induced liver injury with less cytotoxicity *via* activating the antioxidative pathway.

### Transcriptomic Analysis of the Hepatoprotective Effect of Malondialdehyde

**FIGURE 3:**
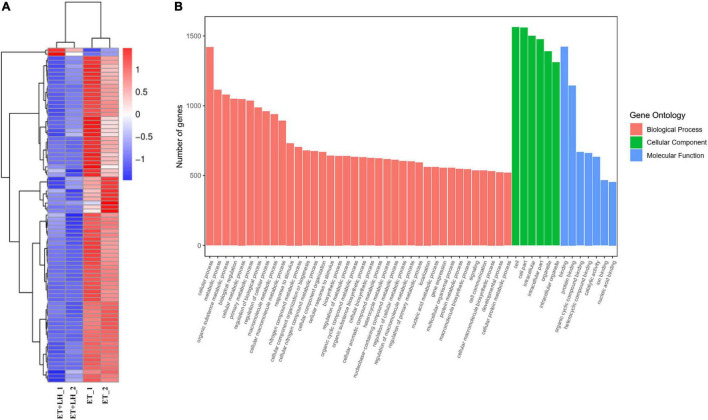
Leonurine hydrochloride (LH) affects the genes expression of LO2 cells. **(A)** Transcriptomic changes of ethanol-treated LO2 cells exposed to LH (10 μM) for 24 h. Cluster heatmap representing differentially expressed genes (DEGs) between the ET group and ET + LH group (*n* = 2). The scaled expression value of each feature is plotted in red–blue color scale. Positive z-score (red) indicates a predicted up-regulated function and negative z-score (blue) is a down-regulated function. **(B)** Gene Ontology (GO) enrichment analysis. Different colors represent different GO: Biological processes (BP), Cellular component (CC) and Molecular functions (MF).

**FIGURE 4:**
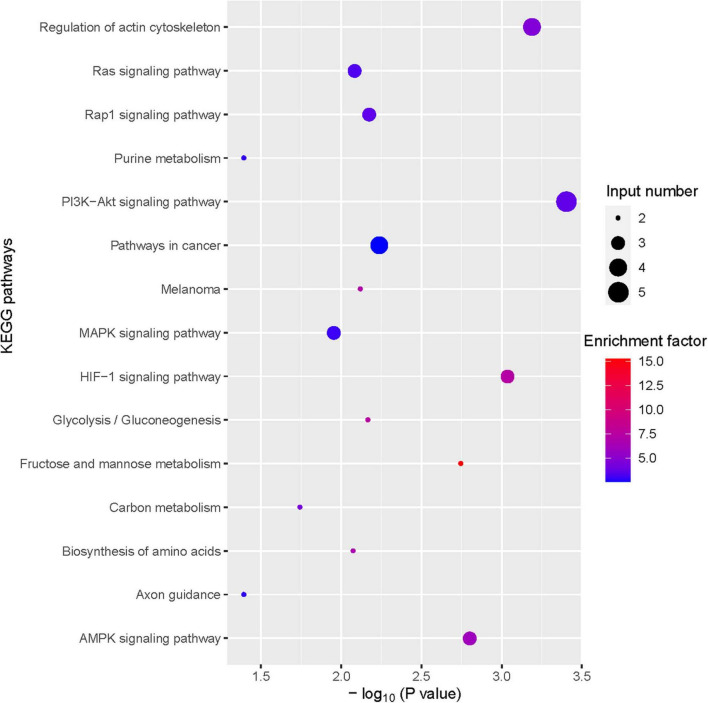
Bubble plot of Kyoto Encyclopedia of Genes and Genomes (KEGG) enrichment of differentially expressed gene (DEGs) (TOP 15).

**FIGURE 5:**
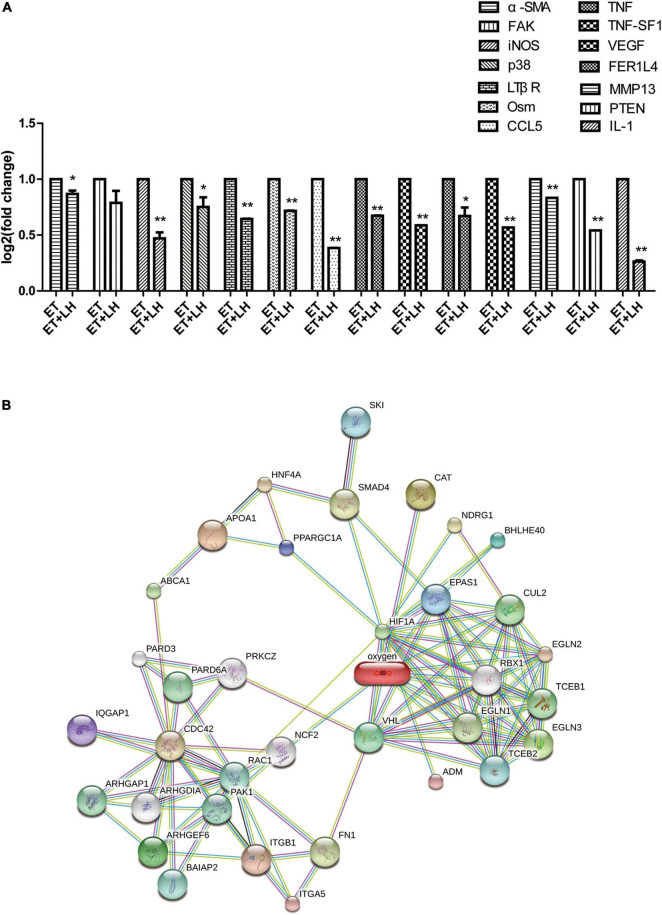
The genes expression changes of Leonurine hydrochloride (LH) against alcoholic liver disease (ALD). **(A)** The expression changes of genes determined by Real-time qPCR (RT-qPCR). Ethanol-treated LO2 cells were exposed to LH (10 μM) for 24 h. Then mRNA was extracted, the mRNA expressions were measured by RT-qPCR. * and ** indicated a significant difference at *p* < 0.05 level, a highly significant difference at *p* < 0.01 level, respectively, between ET group vs. ET + LH group using the independent *t*-test. **(B)** Protein-protein interaction network of LH acting on ALD. Small circles: protein of unknown 3D structure; large circles: some 3D structure is known or predicted.

To further investigate the hepatoprotective mechanism of LH against alcoholic liver injury, transcriptomic analysis was performed after exposure of ethanol-treated LO2 cells to 10 μM LH for 24 h. RNA-seq data revealed that 83 genes were differentially expressed (DEGs) in ET (ethanol-treated LO2 cells) vs. ET + LH (ethanol-treated LO2 cells exposed to LH for 24 h) (Figure 3A). To gain insight into potential mechanisms of LH against ALD, the DEGs were analyzed *via* KEGG and GO database. According to the GO terms, most of the pathways were enriched in Biological Process (BP), Cellular Component (CC), and Molecular Function (MF). The top three processes in BP were the cellular process, metabolic process and organic substance metabolic process, which were involved in the lipid metabolism of hepatocytes. Cell and intra CCs were the most important among CC, while binding, protein binding, metal ion binding and cation binding were identified as the main function in MF (Figure 3B). The KEGG analysis results indicated that four KEGG pathways were mainly involved in PI3K-AKT pathway, regulation of actin cytoskeleton, Hypoxia-inducible factor 1 (HIF-1) pathway, and AMPK pathway (Figure 4). To further validate these results, we performed RT-qPCR assay to evaluate the genes involved in PI3K-AKT, AMPK, regulation of actin cytoskeleton, and HIF-1 signaling pathways. The results indicated that LH can suppress α-SMA, FAK, iNOS, p38, LTβR, Osm, CCL5, TNF, TNF-SF1, VEGF, FER1L4, MMP13, PTEN, and IL-1 mRNA expression in ethanol-treated LO2 cells (Figure 5A). These results are consistent with the KEGG analysis, suggesting that PI3K-AKT pathway, regulation of actin cytoskeleton, HIF-1 pathway, and AMPK pathway are crucial for the hepatoprotective effect of LH against alcoholic liver injury. In order to better understand the mechanism of LH against ALD, a protein–protein interaction network was further constructed by STRING 11.0. All enable sources (“Text mining,” “Experiments,” “Databases,” “Co-expression,” “Neighborhood,” “Gene Fusion,” “Co-occurrence”) were set for active interaction. In this network, the higher degree of interactions were HIF-1α, RAC1, CUL2, and EGLN1 (Figure 5B). These results suggested that these central genes may be the key target genes of LH for protecting liver against oxidative damage.

### Metabolic Analysis of the Hepatoprotective Effect of Malondialdehyde

**FIGURE 6:**
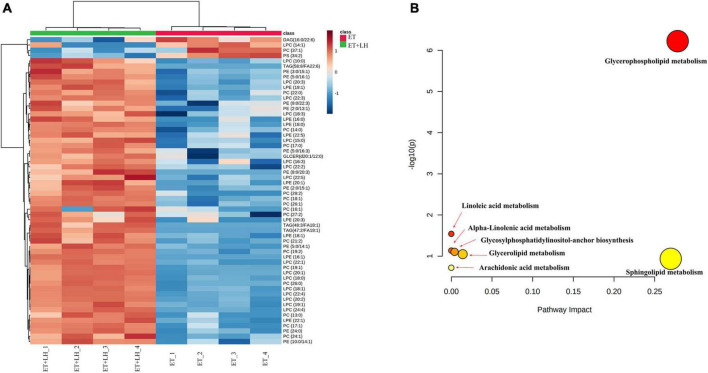
Metabolic analysis of the hepatoprotective effect of Leonurine hydrochloride (LH). **(A)** Heatmaps of identified metabolites between ET and ET + LH with the description of treatments (ethanol-treated cells and those treated with alcohol and LH). The row Z-score or scaled expression value of each feature is plotted in red–blue color scale. Positive z-score (red) indicates a predicted up-regulated function and negative z-score (blue) is a down-regulated function. **(B)** Metabolic pathway analysis by MetaboAnalyst 5.0. Pathway impact value based on the pathway topology analysis. The *x*-axis represents the pathway impact value computed from pathway topological analysis, and the *y*-axis is the-log of the *p*-value obtained from pathway enrichment analysis.

Given the hepatoprotective mechanism of LH against alcoholic liver injury might be involved in the lipid metabolism, we next performed UHPLC-MS/MS to detect the lipid metabolism of ethanol-treated LO2 cells after exposure to LH for 24 h. OPLS-DA was performed to determine the different metabolic profiles between ET and ET + LH groups. The clustering heatmap data revealed that there is a significant difference in 33 metabolites (Phosphatidylcholine, 1-Acyl-sn-glycero-3-phosphocholine, Phosphatidylethanolamine and 1-Acyl-sn-glycero-3-phosphoethanolamine et.al) between the ET and ET + LH groups (Figure 6A). KEGG enrichment analysis of the significantly different metabolites revealed that glycerophospholipid metabolism is the primary metabolic pathway involved in the hepatoprotective effect of LH against alcoholic liver injury (Figure 6B).

## Discussion

Alcohol abuse may trigger oxidative damage and eventually result in ALD (8). Even though ADH inhibitors have been utilized to suppress the progress of ALD, their prognosis is still poor (7, 21). Activation of oxidative stress, GSH depletion, and abnormal lipopolysaccharide (LPS) have been reported to be of great importance in the pathogenesis process of ALD (8, 22). It suggested that restoring oxidative activity and suppressing GSH depletion might be a promising strategy for the therapy development of ALD (23). Many natural products have been utilized as antioxidants against oxidative damage. Alkaloids purified from plant crude extracts have a wide side range of pharmacological activities including *via* antioxidative (24). However, the hepatoprotective activity of these reported alkaloids against ALD is not clear (10–12). In this study, we selected eight alkaloids from a natural product library and found that LH is a potent antioxidant against alcoholic liver injury *via* evaluating the changes of GSH and MDA values in hepatocytes.

Although the underlying mechanism of ALD is complex, accumulated evidence indicates that alcohol abuse can promote the accumulation of fatty acid and stimulate lipogenesis *via* directly regulating transcription of sterol regulatory element-binding protein 1c (SREBP-1c) expression (8, 25). AMPK was identified as an important factor to directly regulate SREBP activity in hepatocytes, thereby repressing lipogenesis synthesis in hepatocytes (8, 26–28). Intriguingly, the transcriptomic analysis revealed that LH exerting its hepatoprotective effect through AMPK pathway. These results are consistent with the previous study showing that activation of AMPK phosphorylation has a protective effect against alcohol consumption-induced liver injury (8, 27). Meanwhile, LH has been reported as a novel hepatoprotective agent against non-alcoholic steatohepatitis mediated *via* AMPK/SREBP1 signaling pathway (29). It suggested that AMPK signaling pathway played a crucial role in hepaprotective effects of LH against ALD. Another study indicated that AMPK pathway was mediated by the activation of PI3K/AKT/eNOS pathway, (30, 31) while the modulation of the PI3K/AKT/eNOS pathway was involved in the protective effects against ALD (32). In the present study, KEGG enrichment analysis revealed that PI3K/AKT pathway was one of the most important pathways in the hepatoprotective effects of LH. To further validate the result, a number of key genes in AMPK and PI3K/AKT pathways were evaluated. The results showed that LH can suppress the gene expression of VEGF, iNOs, p38, α-SMA, Osm, CCL5, MMP13, IL-1, LTPR, FAK, and PTEN in ethanol-treated LO2 cells. These results suggested that LH might protect hepatocytes against alcohol induced liver injury by suppressing AMPK pathway and modulating the activation of PI3K/AKT/eNOS pathway.

Actin is the main constituent of cytoskeleton. Oxidative stress might change the structure of the actin cytoskeleton, thereby leading to apoptosis response (33). The introduction of alcohol would metabolize to acetaldehyde in liver cells. A recent study demonstrated that the reorganization of actin cytoskeleton is associated with acetaldehyde-induced oxidative stress. It indicated that alcohol can induce oxidative stress *via* regulating actin cytoskeleton (34, 35). Previously study revealed that hydrocarbon benzo[a]pyrene (b[a]p), cadmium and copper can induce ROS production and cause disruption of the actin cytoskeleton. Actin cytoskeleton might play an important role in the induction of oxidative stress caused by exogenous substances (36). Although few studies reported the underlying mechanism of the regulation of actin cytoskeleton by LH, the present work suggested that LH may function as a potent antioxidant against ALD *via* the regulation of actin cytoskeleton.

HIF-1 is a transcription factor that regulates oxygen homeostasis. Nath et.al found that the deficiency of HIF-1 can suppress ethanol-induced steatosis in many strains of mice, suggesting that the regulation of HIF-1 pathway might suppress alcoholic fatty liver damage (37). In our study, we noticed that HIF-1 pathway is important for remedying ALD. This result suggested that the hepatoprotective effect of LH might be partly attributed to the down regulation of HIF-1 pathway. Activation of HIF-1 pathway can attenuate TNF signaling pathways, while suppression of PI3K/AKT pathway would activate TNF signaling pathway (38, 39). It suggested that the hepatoprotective activity of LH might be associated with the reduction of TNF signaling pathway. TNF receptor associated factors act as adaptors to link LTβR with NFκB. Intriguingly, we noticed that LH could suppress LTβR, TNF, TNF-SF1, and FER1L4 mRNA in ethanol-treated LO2 cells. Similar studies display that LH can suppress inflammatory responses *via* NF-κB and PI3K/AKT signaling pathway (40, 41). These results indicated that LH can attenuate TNF signaling pathway and might be associated with the modification of HIF-1, AMPK, and PI3K/AKT pathways. A protein–protein interaction network analysis showed that HIF-1α, RAC1, CUL2, and EGLN1 were the key target genes of LH. These results indicated that these principal target genes of LH might be important for protecting liver from oxidative damage.

Leonurine hydrochloride functions as a hepatoprotective agent against liver injury by improving intracellular lipid accumulation. The hepatic lipid metabolism is regulated by various transcription factors and pathways including PI3K and its downstream kinases such as AKT, PTEN, atypical protein kinase C (aPKC) and mTOR (29, 42, 43). Increasing phosphatidylcholine is a potential strategy for acute liver injury therapy (44). Hepatic phosphatidylethanolamine methyltransferase activity is reduced by ethanol and increased by phosphatidylcholine (45). To evaluate the underlying mechanism of LH on hepatic lipid metabolism, we determined the lipid metabolisms of LH in ethanol-treated LO2 cells. The clustering heatmap data and KEGG enrichment analysis revealed that Phosphatidylcholine, 1-Acyl-sn-glycero-3-phosphocholine, Phosphatidylethanolamine and 1-Acyl-sn-glycero-3-phosphoethanolamine are significantly different between the ET and ET + LH groups, while glycerophospholipid metabolism is the primary metabolic pathway involved in the hepatoprotective effect of LH against alcoholic liver injury. Glycerophospholipid metabolism pathway is essential for oxidative stress and can stable cell membranes to relieve the cell injury *via* hypoxic stress (46, 47). A previous study revealed that ethanol exposure can alter glycerophospholipids metabolism in liver tissues, (48) while we found that LH can restore the glycerophospholipids metabolites, suggesting that the hepatoprotective of LH against ALD might be partly attributed to the activation of glycerophospholipid metabolism pathway. Raspberry anthocyanin (RA) is an antioxidant that can promote the recovery of glycerophospholipid metabolism (46). Blackberry anthocyanins and blueberry anthocyanins can significantly attenuate TNF-α, IL-6, and NFκB genes expression and regulate glycerophospholipid metabolism to repair oxidative damage *via* accelerating energy expenditure (49). Our results are consistent with the finding in the previous literatures. Our results suggested that LH can attenuate TNF signaling pathway to protect cells against oxidative stress and that glycerophospholipid metabolism is a primary metabolic pathway involved in the hepatoprotective effect of LH against alcoholic liver injury.

In summary, this study has identified LH as a promising and low hepatotoxic antioxidant compound against ALD. In terms of mechanism, LH significantly modulated genes involved in the regulation of PI3K-AKT, AMPK, actin cytoskeleton, and HIF-1 signaling pathways. Moreover, the expression patterns of genes involved in the glycerophospholipid metabolism were also affected in this process. Altogether, this study suggested that LH could be a promising candidate compound for further mechanism study and could be utilized as a novel antioxidant alkaloid scaffold against ALD. Notably, to better understand the mechanism and pharmacokinetic (PK) behavior of LH, the investigation of oral absorption/bioavailability of LH might be highly required under ALD *in vivo* model in the future.

## Data Availability Statement

The original contributions presented in this study are included in the article/Supplementary Material, further inquiries can be directed to the corresponding author. Raw reads of the transcriptome project have been deposited in NCBI’s BioProject accession number PRJNA822708.

## Author Contributions

ZG: conceptualization, resources, supervision, project administration, and funding acquisition. K-JW: methodology, formal analysis, data curation, and writing—original draft preparation. P-PL: software. P-PL, M-YC, and XL: validation. M-XZ: investigation. K-JW, XL, and QY: writing—review and editing. LX: visualization. All authors have read and agreed to the published version of the manuscript.

## Conflict of Interest

The authors declare that the research was conducted in the absence of any commercial or financial relationships that could be construed as a potential conflict of interest.

## Publisher’s Note

All claims expressed in this article are solely those of the authors and do not necessarily represent those of their affiliated organizations, or those of the publisher, the editors and the reviewers. Any product that may be evaluated in this article, or claim that may be made by its manufacturer, is not guaranteed or endorsed by the publisher.

## Footnotes

1
http://www.heatmapper.ca/expression/

2
www.metaboanalyst.ca
