# Supplementary material for: The Hepatoprotective Effect of Leonurine Hydrochloride Against Alcoholic Liver Disease Based on Transcriptomic and Metabolomic Analysis
Source: Front Nutr. 2022 Jul 7;9:904557. doi: 10.3389/fnut.2022.904557 (PMC9301321; doi:10.3389/fnut.2022.904557)
Supplement: Supplementary file 1 [file Table_1.docx]

Supplementary Material

**The hepatoprotective effect of leonurine hydrochloride against alcoholic liver disease based on transcriptomic and metabolomic analysis**

**Ke-Jia Wu ^1†^, Pin-Pin Liu ^1†^, Meng-Yuan Chen ^1^, Meng-Xin Zhou ^1^, Xin Liu ^1^, Qing Yang ^1^, Lin Xu ^1^, and Zhiyong Gong ^1,^***

^1^ Key Laboratory for Deep Processing of Major Grain and Oil of Ministry of Education, Wuhan Polytechnic University, Wuhan, China

^†^ These authors contributed equally to this work

**Supplementary Table**

Table 1. Primer sequences of RT-qPCR target genes.([1-6](#_ENREF_1))

| Gene | Primer Sequence (Forward) | Primer Sequence (Reverse) |
| --- | --- | --- |
| α-SMA | GTGACTACTGCCGAGCGTG | ATAGGTGGTTTCGTGGATGC |
| FAK | AAAGCAGTAATGAGCCAACCAC | TGAGGCGAAATCCATAGCAG |
| iNOs | GCAGAATGTGACCATCATGG | ACAACCTTGGTGTTGAAGGC |
| p38 | ACCACGACCCTGATGATGAGC | TAGGTCAGGCTCTTCCATTCGT |
| LTβR | AGAAAGAGGACCAGCGACAG | GCAGCTCCAGGTACCTCCTA |
| Osm | TGCTCAGGATGAGGAGACTG | AGCTGTGTCACCCCTGAGAG |
| CCL5 | GTCGTCTTTGTCACCCGAAAG | TCCCGAACCCATTTCTTCTCT |
| TNF | AACTAGTGGTGCCAGCCG | CTTCACAGAGCAATGACTCC |
| TNF-SF1 | TCGTGTCTCCCATAACAG | GATCAACGATCTCACCAGGC |
| VEGF | GCTGCAATGATGAAGCCCTG | TTAACTCAAGCTGCCTCGCC |
| FER1L4 | CCGTGTTGAGGTGCTGTTC | GGCAAGTCCACTGTCAGATG |
| MMP13 | CCAGAACTTCCCAACCAT | ACCCTCCATAATGTCATACC |
| PTEN | GTTTACCGGCAGCATCAAAT | CCCCCACTTTAGTGCACAGT |
| IL-1 | CGCAGCAGCACATCAACAAGA | TGTCCTCATCCTGGAAGGTCC |
| β-Actin | GGATGCAGAAGGAGATCACTG | CGATCCACACGGAGTACTTG |

1. M. Qiao, J. Yang, Y. Zhu, Y. Zhao and J. Hu: Transcriptomics and proteomics analysis of system-level mechanisms in the liver of apigenin-treated fibrotic rats. *Life Sciences*, 248, 117475 (2020)

2. L. D’Ignazio, M. Batie and S. Rocha: TNFSF14/LIGHT, a non-canonical NF-κB stimulus, induces the HIF pathway. *Cells*, 7(8), 102 (2018)

3. P. Krause, S. P. Zahner, G. Kim, R. B. Shaikh, M. W. Steinberg and M. Kronenberg: The tumor necrosis factor family member TNFSF14 (LIGHT) is required for resolution of intestinal inflammation in mice. *Gastroenterology*, 146(7), 1752-1762. e4 (2014)

4. H. Ding, J. Chen, J. Qin, R. Chen and Z. Yi: TGF-β-induced α-SMA expression is mediated by C/EBPβ acetylation in human alveolar epithelial cells. *Molecular Medicine*, 27(1), 1-12 (2021)

5. A. Abdelbaset-Ismail, M. Cymer, S. Borkowska-Rzeszotek, K. Brzeźniakiewicz-Janus, P. Rameshwar, S. S. Kakar, J. Ratajczak and M. Z. Ratajczak: Bioactive phospholipids enhance migration and adhesion of human leukemic cells by inhibiting heme oxygenase 1 (HO-1) and inducible nitric oxygenase synthase (iNOS) in a p38 MAPK-dependent manner. *Stem cell reviews and reports*, 15(1), 139-154 (2019)

6. H.-A. Cai, L. Huang, L.-J. Zheng, K. Fu, J. Wang, F.-D. Hu and R.-Y. Liao: Ginsenoside (Rg-1) promoted the wound closure of diabetic foot ulcer through iNOS elevation via miR-23a/IRF-1 axis. *Life sciences*, 233, 116525 (2019)
